# Dual-loss of PBRM1 and RAD51 identifies hyper-sensitive subset patients to immunotherapy in clear cell renal cell carcinoma

**DOI:** 10.1007/s00262-024-03681-x

**Published:** 2024-04-12

**Authors:** Ziyang Xu, Wenbin Jiang, Li Liu, Youqi Qiu, Jiahao Wang, Siyuan Dai, Jianming Guo, Jiejie Xu

**Affiliations:** 1 Department of Urology, Zhongshan Hospital, Fudan University, Shanghai, 200032 China; 2 NHC Key Laboratory of Glycoconjugate Research, Department of Biochemistry and Molecular Biology, School of Basic Medical Sciences, Fudan University, Shanghai, 200032 China

**Keywords:** Polybromo 1, RAD51, Homologous recombination deficiency, Clear cell renal cell carcinoma, Immunotherapy

## Abstract

**Background:**

Homologous recombination deficiency (HRD), though largely uncharacterized in clear cell renal cell carcinoma (ccRCC), was found associated with RAD51 loss of expression. PBRM1 is the second most common mutated genes in ccRCC. Here, we introduce a HRD function-based PBRM1-RAD51 ccRCC classification endowed with diverse immune checkpoint blockade (ICB) responses.

**Methods:**

Totally 1542 patients from four independent cohorts were enrolled, including our localized Zhongshan hospital (ZSHS) cohort and Zhongshan hospital metastatic RCC (ZSHS-mRCC) cohort, The Cancer Genome Atlas (TCGA) cohort and CheckMate cohort. The genomic profile and immune microenvironment were depicted by genomic, transcriptome data and immunohistochemistry.

**Results:**

We observed that PBRM1-loss ccRCC harbored enriched HRD-associated mutational signature 3 and loss of RAD51. Dual-loss of PBRM1 and RAD51 identified patients hyper-sensitive to immunotherapy. This dual-loss subtype was featured by M1 macrophage infiltration. Dual-loss was, albeit homologous recombination defective, with high chromosomal stability.

**Conclusions:**

PBRM1 and RAD51 dual-loss ccRCC indicates superior responses to immunotherapy. Dual-loss ccRCC harbors an immune-desert microenvironment but enriched with M1 macrophages. Dual-loss ccRCC is susceptible to defective homologous recombination but possesses high chromosomal stability.

**Supplementary Information:**

The online version contains supplementary material available at 10.1007/s00262-024-03681-x.

## Introduction

Renal cell carcinoma (RCC) is one of the top ten most frequent cancer types worldwide, affecting over 400,000 patients per year [1]. Among RCC, clear cell renal cell carcinoma (ccRCC) is the most common subtype, accounting for 70–80% of cases [2, 3]. Immune checkpoint blockade (ICB) therapies, including dual ICB and combinations with tyrosine kinase inhibitors (TKIs), have emerged as the first-line strategy for advanced ccRCC. While durable responses to ICB have been observed in responders, resistance commonly manifests in many others [3, 4]. Identifying patients benefit from immunotherapy or not remains a crucial unmet need to maximize efficacy without exacerbating toxic effects. However, widely explored biomarkers, like programmed cell death ligand 1(PD-L1) and tumor mutation burden (TMB), fail to predict responses to ICB in ccRCC [5–7]. Single biomarker based on certain genomic feature or gene expression, either mRNA or protein level, may limit predictive power due to unidimensional therapeutical information [8]. Thus, combination of multiple non-interfering features might be a successful solution to guide therapeutic stratification.

PBRM1 encodes BAF180, a functional subunit of SWI/SNF chromatin remodeling complex [9, 10]. On genomic background, PBRM1 is mutated in about 40% of all ccRCC cases [4, 11]. Previous studies reported that PBRM1 mutations, more than 90% of which are truncating mutations (including frameshift indels, nonsense, and splice site mutations), coincide with PBRM1 loss-of-function [4, 11]. Nearly, all PBRM1 mutations were found in the context of loss of heterozygosity on 3p, with the allelic loss at 3p21 [11]. Regarding ccRCC, PBRM1-loss tumors tend to present nonimmunogenic tumor phenotypes [12], elevated replications stress, DNA double-strand break (DSB) and genomic instability [10]. Several preclinical or retrospective studies have shown that PBRM1 seems to be predictive of immunotherapeutic responses [4, 7, 13], but to date still controversial and unevaluated in the clinical decision-making process [8], which is likely due, in part, to the fact that PBRM1 alone lack the capacity to define a homogenous molecular ccRCC subtype.

DNA damage repair (DDR) deficiency has important implications for tumor evolution and responses to therapies [2, 14]. Homologous recombination (HR) is the main mechanism involved in DNA double-strand break repair [15]. HRD can confer specific genomic phenotypes and therapeutical responses in different cancer types [16, 17]. However, the majority of HRD cases in ccRCC do not have an obvious hereditary explanation, and underlying damage is more often obscure [2]. An improved understanding of HRD and its correlation with therapeutic responses in ccRCC is of significant importance.

RAD51, a key protein in the homologous recombinational DNA repair (HR) pathway, is regarded as the marker of HR functionality [16]. Substantial evidence indicates that RAD51 fails to accumulate in defected HR pathways [17]. RAD51 has proved to be a predictive marker to therapeutic responses in various cancers, including platinum-based chemotherapy benefit in breast cancer [16], esophageal squamous cell carcinoma [18], PARP inhibitor benefit in pancreatic cancer [19] and prostate cancer [20]. But, few studies converged on the association between HRD-associated RAD51 and therapeutic responses in ccRCC [2].

In this study, we tempted to approximate the HRD status of ccRCC based on a PBRM1-RAD51 classification and link it to immunotherapy responses. A highly homologous recombination deficient subgroup, featured as the dual-loss of PBRM1 and RAD51, was found with superior responses to immunotherapy. Further, a comprehensive immuno-genomic atlas of this subgroup was evaluated. Overall, our findings dissected a specific PBRM1-RAD51 dual-loss ccRCC subtype hyper-sensitive to immunotherapy and revealed its specific immuno-genomic profiles, which may shed light on precise ccRCC treatment decisions.

## Methods

### Patient inclusions

Our localized population included patient-level data from two independent cohorts: Zhongshan hospital (ZSHS) cohort (*n* = 292) and Zhongshan hospital metastatic RCC (ZSHS-mRCC) cohort (*n* = 128).

Zhongshan hospital (ZSHS) cohort enrolled 292 patients diagnosed with ccRCC from Zhongshan Hospital, Fudan University (Shanghai, China). ccRCC diagnoses were based on clinical criteria or histopathology. Patients were enrolled between January 2005 and June 2007 on the basis of following criteria: (i) informed consent, (ii) ccRCC (pathologically proven) patients treated with nephrectomy and (iii) no history of other malignancies, (iv) available for formalin-fixed paraffin embedded specimens. None of the patients have received neoadjuvant or adjuvant systemic therapy. In this study, 58 patients were excluded due to TMA detachment or unassessable PBRM1/ RAD51 staining. Ultimately, 234 patients were eligible for further analysis (Supplementary Fig. 1). The clinicopathological characteristics of the patients from ZSHS cohort are summarized in Supplementary Table 1.

Zhongshan metastatic renal cell carcinoma (ZSHS-mRCC) cohort involved 128 patients diagnosed with mRCC from Zhongshan Hospital, Fudan University (Shanghai, China), treated with ICB or TKI alone (Supplementary Fig. 1). Zhongshan immune checkpoint blockade (ZSHS-ICB) cohort is consisted of 62 patients treated with anti-PD1 or anti-PD1 plus TKI at Zhongshan Hospital, Fudan University (Shanghai, China) between July 2016 and July 2023. Zhongshan tyrosine kinase inhibitors (ZSHS-TKI) cohort is consisted of 66 patients treated with TKI treatment alone at Zhongshan Hospital, Fudan University (Shanghai, China) between April 2012 and July 2023. Patients were selected on the basis of following criteria: (i) informed consent, (ii) RCC (pathologically proven) patients treated with nephrectomy and (iii) available for formalin-fixed paraffin embedded specimens. ZSHS-ICB cohort was composed of 58 ccRCC patients, two papillary RCC patients, one poorly differentiated RCC and one chromophobe renal cell carcinoma. ZSHS-TKI cohort was composed of 59 ccRCC patients, five papillary RCC patients, one renal medullary carcinoma and one MiT family translocation renal cell carcinoma. Clinical response to immunotherapy and TKI was evaluated by Response Evaluation Criteria in Solid Tumors (RECIST) V.1.1 [21]. The clinicopathological characteristics of the patients from ZSHS-ICB cohort are summarized in Supplementary Table 2. The immune-related adverse events (irAEs) of the patients from ZSHS-ICB cohort are summarized in Supplementary Table 3.

### Public data sets

Patients from another two public datasets, including The Cancer Genome Atlas (TCGA) cohort (*n* = 530) and the CheckMate cohort (*n* = 592) (Supplementary Fig. 1), were enrolled.

TCGA cohort is composed of 530 patients, with both clinical information and mRNA expression data available in TCGA kidney clear cell renal cell carcinoma (KIRC) cohort. In this study, 198 patients were excluded due to unavailable genomic data. Ultimately, 332 patients were eligible for further analysis (Supplementary Fig. 1).

CheckMate 025 is a randomized phase III trial following phase I and phase II studies conducted in a similar clinical setting, namely CheckMate 009 and CheckMate 010 [3, 4]. We obtained individualized data of totally 592 patients enrolled in these three trials from previous studies [4, 22]. In this study, 375 patients were excluded due to unavailable genomic or transcriptomic data. Ultimately, 217 patients were eligible for further analysis (Supplementary Fig. 1). From the research led by Toni K. Choueiri [4], clinical, genomic and normalized mRNA data from CheckMate 009/010/025 were obtained. From the research led by Sabina Signoretti [22], mRNA expression data from CheckMate 025 cohort in original TPM form were obtained for further investigation of the distribution of RAD51.

### Processing of genomic and transcriptomic data

The mutational signature 3 calculation was carried out with SigMA algorithm [23]. For analysis of TCGA tumors, the in-built multivariate classifiers with data parameter “tcga_mc3” were used. Similarly, for CheckMate WES data, Sig3 predictions were performed with the in-built multivariate classifiers with data parameter “seq-cap”. We report results for signatures 1 (5’methylcytosine deamination), 3 (homologous recombination defect), 5 (T > C substitution) and 2 and 13 (apolipoprotein B mRNA editing enzyme, catalytic poly- peptide-like) owing to their overwhelming  high mutational frequencies [24]. CNApp was used to quantify individual CNA burdens and to generate the broad and focal CNA scores [25]. The infiltration of immune cells was calculated by CIBERSORTx [26]. Suppression score, infiltration score, immune-related signatures and pathway activation scores were calculated by the single sample gene set enrichment analysis (ssGSEA) algorithms based on related genes expression [27]. Moreover, immune scores were evaluated by ESTIMATE [28]. The canonical oncogenic pathways alternations were computed using “maftools” package [29]. The involved signatures for ssGSEA were defined by previous studies (Supplementary Table 2). In TCGA cohort, TCR-related data, other genomic-related data and CNA signature data were downloaded from previous studies [14, 30] (Supplementary Table 2).

On the basis of positively skewed distributions, RAD51 loss was defined as patients with RAD51 loss of expression (lower than 67th quantiles mRNA expression) in TCGA and CheckMate cohort (Supplementary Fig. 3A, B). It has been reported that PBRM1 mutation accompanies PBRM1 protein loss and decreased PBRM1 mRNA expression, and immunohistochemistry assays could report on their genetic status at high accuracy [31]. Thus, in TCGA and CheckMate cohort, PBRM1 loss was defined as patients with PBRM1 mutation.

### PBRM1 and RAD51 loss classification criteria

In ZSHS and ZSHS-mRCC cohorts, IHC staining for PBRM1 was performed according to previous protocols by two pathologists who were blind to the clinical information with NanoZoomer-XR [32]. PBRM1 loss was defined as the intranuclear staining loss of PBRM1 in tumor cells, as previously described (representative IHC images, Supplementary Fig. 2B) [31, 33].

In TCGA and CheckMate cohorts, patients were classified into RAD51 loss and non-loss subgroups based on RAD51 mRNA expression level. In ZSHS cohort and ZSHS-mRCC cohorts, patients were classified based on the RAD51 H-score. Positively skewed distribution was observed in all three cohorts (Shapiro–Wilk Normality Test *P* < 0.001, *P* < 0.001 and *P* < 0.001 respectively; Supplementary Fig. 3A–C). Meanwhile, according to previous observations, 67% of ccRCC exhibit features of homologous recombination deficiency, with RAD51 loss of expression as a feature [34, 35]. On the basis of such evidence, the cutoff value of RAD51 status was defined as patients with 67th quantiles of RAD51 expression. In ZSHS and ZSHS-mRCC cohorts, RAD51 loss was defined as low RAD51 nuclear immunoreactivity (lower than 67th quantiles, at 122.43, representative IHC images, Supplementary Fig. 2A) [36, 37].

### Immunohistochemistry and assay methods

The antibodies used for IHC staining are summarized in Supplementary Table 3. In ZSHS and ZSHS-mRCC cohorts, IHC staining for RAD51 was performed according to previous protocols and evaluated as the histoscore (H-score) by two pathologists who were blind to the clinical information with NanoZoomer-XR [32]. H-score equals the percentage of positive tumor cell nuclei multiplied by intensity of staining (1, weak; 2, moderate; and 3, strong). The final H-score ranges from 0 (minimum) to 300 (maximum).

### Statistical analysis

Kaplan–Meier analysis and log-rank test were performed to conduct survival analysis. Univariate analysis was conducted using the Cox proportional hazards regression model. Pearson’ s Chi-squared test was applied to detect categorical variables. Statistical *P* Values were computed using Mann–Whitney U test and Kruskal–Wallis test. *P* value less than 0.05 was considered statistically significant. All statistical analysis were conducted using IBM SPSS Statistics 26.0 and R software 4.1.2.

## Results

### PBRM1-loss ccRCC harbors enriched HRD-associated mutational signature 3

**Fig. 1:**
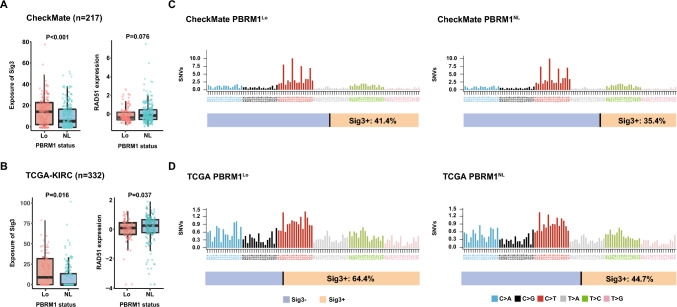
PBRM1-loss ccRCC harbors enriched HRD-associated mutational signature 3 and HRD-associated RAD51 loss. Boxplots showing the association between PBRM1 status and HRD-associated mutational signature 3 and normalized RAD51 expression in **A** CheckMate cohort and in **B** TCGA cohort. RAD51 mRNA expression was normalized by Z-score. Data were analyzed by Mann–Whitney U test. Average mutational spectra of different PBRM1 status ccRCC by SigMA in **C** CheckMate cohort and in **D** TCGA cohort. The horizontal bar below shows the fractions of Sig3+ patients. *P* ≤ 0.05 was considered statistical significance. *Lo* Loss; *NL* Non-loss

Homologous recombination deficiency, though existing in a large number of ccRCC patients, remains poorly defined beyond HR-related germline alternations [2]. In both CheckMate and TCGA cohort, we observed enriched HRD-associated mutational signature 3 (*P* < 0.001 and *P* = 0.016 respectively; Fig. 1A, B) as well as RAD51 loss of expression (*P* = 0.076 and *P* = 0.037 respectively; Fig. 1A, B), a HR functional marker, in PBRM1-loss ccRCC. In addition, IHC results from localized cohorts confirmed that PBRM1-loss ccRCC owned the characteristic enrichment of RAD51 loss (*P* = 0.013; Supplementary Fig. 2C). Taken together, these data suggest that PBRM1-loss ccRCC is associated with an elevated HRD status.

### RAD51 loss ccRCC demonstrates better responses to immunotherapy

**Fig. 2:**
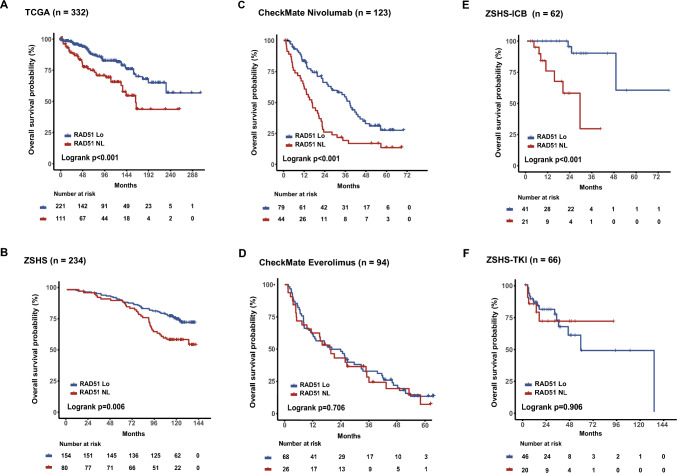
HRD-associated RAD51 loss predicts prognosis and responsiveness to immunotherapy. Kaplan–Meier analysis of patients’ prognosis with RAD51 loss or RAD51 non-loss ccRCC in **A** TCGA cohort and **B** ZSHS cohort. Kaplan–Meier analysis of therapeutic responses with RAD51 loss or RAD51 non-loss ccRCC treated with **C** Nivolumab and **D** Everolimus in CheckMate cohort. Kaplan–Meier analysis of therapeutic responses with RAD51 loss or RAD51 non-loss ccRCC treated with **E** immunotherapy and **F** TKI alone in ZSHS-mRCC cohort. Log-rank test was conducted for Kaplan–Meier curves. *P* ≤ 0.05 was considered statistical significance. *Lo* Loss; *NL* Non-loss

We then sought to investigate the association between RAD51 status and clinical features. Based on its positively skewed distribution, RAD51 loss was defined as those below the 67th quantiles of RAD51 expression in mRNA sequencing cohorts (Supplementary Fig. 3). We observed that RAD51-loss was significantly correlated with lower TNM stages in TCGA cohort (*P* < 0.001; Supplementary Fig. 4A). Kaplan–Meier curves showed that RAD51 loss was associated with superior overall survival (OS) (*P* < 0.001; Fig. 2A). To further validate the results, we then explored ZSHS cohort and applied the same cutoff to ZSHS cohort and found that RAD51 loss was associated with superior OS as well (*P* = 0.006; Fig. 2B).

Further, the association of RAD51 and ICB responses was investigated in CheckMate cohort. Patients with RAD51 loss derived survival benefits from immunotherapy (Hazard Ratio [HR]: 0.498, 95% Confidential interval [CI] 0.322–0.771, log-rank *P* < 0.001; Fig. 2C), but not Everolimus (Hazard Ratio [HR]: 0.946, 95% Confidential interval [CI] 0.583–1.534, log-rank *P* = 0.706; Fig. 2D). Survival analysis in ZSHS-mRCC cohort was performed to further validate such findings (*n* = 128). Of the 62 patients treated with immunotherapy, patients with RAD51 loss had significant survival benefits from immunotherapy (Hazard Ratio [HR]: 0.076, 95% Confidential interval [CI] 0.015–0.378, log-rank *P* < 0.001; Fig. 2E), while of the 66 patients treated with TKI alone, no differences were found (Hazard Ratio ([HR]: 1.056, 95% Confidential interval [CI] 0.375–2.974, *P* = 0.906; Fig. 2F).

### Dual-loss of PBRM1 and RAD51 identifies patients hyper-sensitive to immunotherapy

Pursuing the idea that the combination of RAD51 and PBRM1 might help define different ccRCC subtypes endowed with discordant prognosis and responses to immunotherapy, we assessed whether subtypes built on their status could stratify prognosis and the efficacy of ICB. In both TCGA and ZSHS cohort, PBRM1 and RAD51 dual-loss patients featured the best prognosis (*P* = 0.006 and *P* = 0.017, respectively; Supplementary Fig. 4B, C). Dual-loss patients also possessed the lowest TNM stages (*P* = 0.015; Supplementary Fig. 4D).

**Fig. 3:**
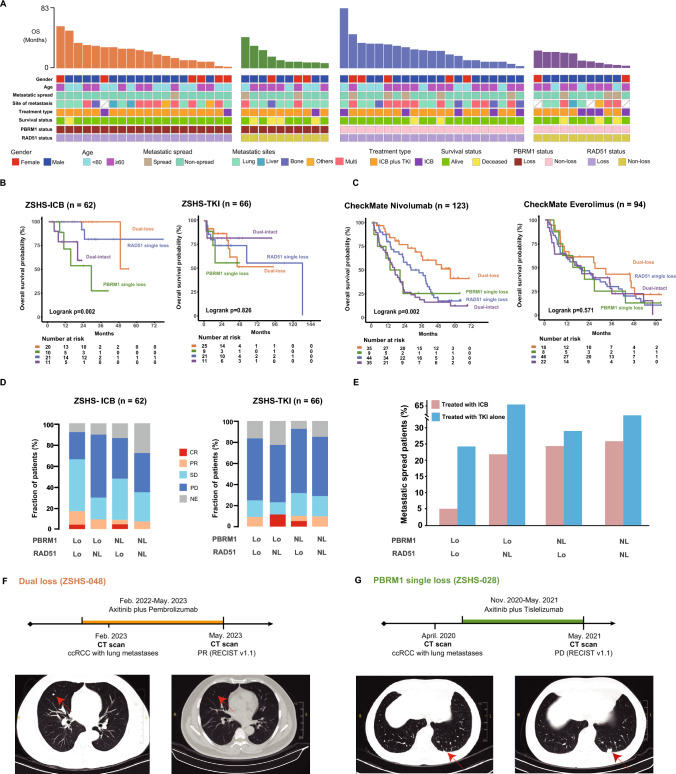
Dual-loss of PBRM1 and RAD51 identifies patients vulnerable to immunotherapy.** A** Heatmap of clinical and molecular features based on PBRM1/RAD51 status in the ZSHS-ICB cohort. **B** Kaplan–Meier analysis demonstrating therapeutic responses of PBRM1 and RAD51 dual-loss ccRCC patients in ZSHS-ICB cohort and ZSHS-TKI cohort. **C** Kaplan–Meier analysis demonstrating therapeutic responses of PBRM1 and RAD51 dual-loss ccRCC patients treated with Nivolumab and Everolimus in CheckMate cohort. **D** Association of the objective response rates with PBRM1/RAD51 status in ZSHS-ICB and ZSHS-TKI cohorts. **E** Bar charts showing the fraction of metastatic spread patients within each subgroup in ZSHS-ICB and ZSHS-TKI cohorts. **F** CT scans showing typical partial response (PR) imaging alteration of a patient from ZSHS-mRCC cohort after Axitinib plus Pembrolizumab treatment. The red arrow highlights a target lesion and its evolution. **G** CT scans showing typical progressive disease (PD) imaging alteration of a patient from ZSHS-mRCC cohort after Axitinib plus Tislelizumab treatment. The red arrow highlights a target lesion and its evolution. Log-rank test was conducted for Kaplan–Meier curves. *P* ≤ 0.05 was considered statistical significance. *Lo* Loss; *NL* Non-loss

In ZSHS-mRCC cohort, we assessed the relationship of PBRM1/RAD51 status and corresponding clinical annotations (Fig. 3A). In terms of overall survival probability, PBRM1/RAD51 dual-loss patients featured highest sensitivity to immunotherapy (log-rank *P* = 0.002), but not to TKI (log-rank *P* = 0.826; Fig. 3B). In order to ascertain whether the enhanced ICB responses in dual-loss ccRCC is attributed to its prognostic value, we performed a multivariate Cox regression with the inclusion of clinicopathological factors including age, gender, treatment types and WHO/ISUP grades in ZSHS-ICB cohort, and the dual-loss status was found as an independent stratification factor for ICB responses (Supplementary Fig. 5). Further, we validated the hypothesis in the CheckMate cohort. The PBRM1-RAD51 model dwarfed PBRM1 or RAD51 alone in identifying ICB responses (Supplementary Fig. 6A, B). PBRM1 and RAD51 dual-loss patients featured the best response to Nivolumab (Hazard Ratio [HR]: 0.391, 95% Confidential interval [CI] 0.219–0.695, log-rank *P* = 0.002; Fig. 3C), but not Everolimus (Hazard Ratio [HR]: 1.061, 95% Confidential interval [CI] 0.409–2.755, log-rank *P* = 0.571; Fig. 3C).

Within PBRM1 and RAD51 dual-loss tumors, complete response (CR)/partial response (PR) was achieved in 4 patients [20.0%], and 9 patients [29.4%] had stable disease (SD), overwhelming other subgroups in ZSHS-ICB cohort, especially compared with PBRM1 single-loss tumors (Fig. 3D). Patients with de novo metastasis tend to suffer metastatic spread, leading to largely reduced survival and limited opportunity for treatment [13, 22]. PBRM1-loss patients (both in dual-loss and PBRM1 single-loss subgroups) had significantly lower post-treatment metastatic spread when treated with immunotherapy than TKI alone (Fig. 3E). Specifically, PBRM1 and RAD51 dual-loss ccRCC treated with immunotherapy had the lowest probability (one in 20 patients, [5.0%]) of post-treatment metastatic spread among subgroups (Fig. 3E).

Significant therapeutic response was observed in a clinical case of a 46-year-old patient (ZSHS-048) diagnosed with metastatic ccRCC harboring dual-loss of PBRM1 and RAD51 (Fig. 3F). Following Axitinib plus Pembrolizumab, the largest lung metastatic site displayed remission as indicated by CT scans (Fig. 3F). Another 55-year-old patient (ZSHS-028) from ZSHS cohort with PBRM1 single-loss failed to receive long-term clinical response after Axitinib plus Tislelizumab, whose lung metastatic lesion achieved progressive disease (PD) after immunotherapy (Fig. 3G). Patients with single PBRM1 loss exhibited significantly worse ICB benefits compared with the dual-loss counterparts.

### Dual-loss of PBRM1 and RAD51 ccRCC harbors immune-desert but effective microenvironment enriched with M1 macrophage infiltration

**Fig. 4:**
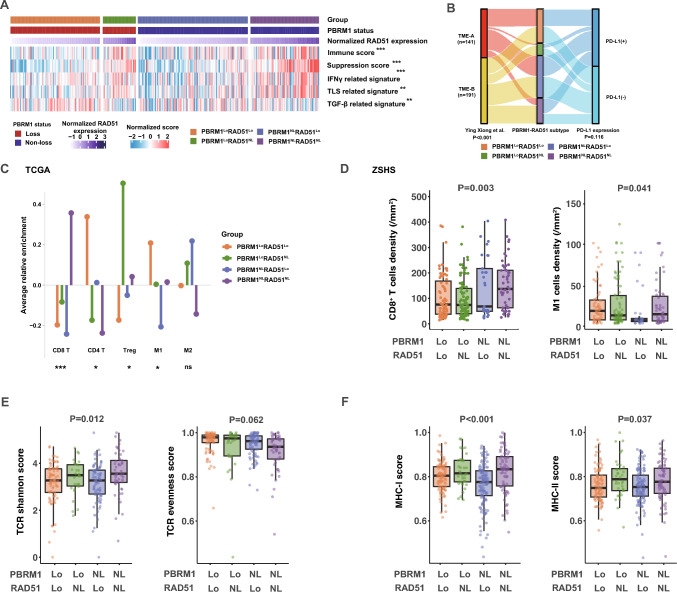
Dual-loss of PBRM1 and RAD51 ccRCC harbors immune-desert but effective microenvironment enriched for M1 polarization. **A** Heatmap showing the abundance pathways signature score of immune relevant pathways in TCGA cohort. Data were analyzed by Kruskal–Wallis test. **B** Sankey diagram showing the flow of RAD51-PBRM1 subtypes to TME-A/B distribution and PD-L1 mRNA expression levels. Data were analyzed by Pearson’s Chi-squared test. **C** Dot chart illustrating different immune cell enrichment scores calculated by ssGSEA across subgroups in TCGA cohort. Data were analyzed by Kruskal–Wallis test. **D** Boxplots demonstrating the CD8^+^ T cells and M1 cells infiltration across subgroups in ZSHS cohort. Data were analyzed by Kruskal–Wallis test. **E** Boxplots depicting the abundance of MHC-I/ II scores across subgroups in TCGA cohort. Data were analyzed by Kruskal–Wallis test. **F** Boxplots depicting the abundance of TCR relevant scores across subgroups in TCGA cohort. Data were analyzed by Kruskal–Wallis test. *P ≤ 0.05; **P ≤ 0.01; ***P ≤ 0.001. *P* ≤ 0.05 was considered statistical significance. *Lo* Loss; *NL* Non-loss; *TGFβ* Transforming growth factor beta; *TLS* Tertiary lymphoid structure; *IFNγ* Interferon gamma

We further depicted the immuno-genomic repertoire of PBRM1 and RAD51 dual-loss ccRCC. Intriguingly, ICB-sensitive dual-loss ccRCC did not exhibit presumed indicators of immunotherapy sensitivity and instead manifested a non-infiltrated microenvironment featured by lower immune scores, suppression scores, immune-related pathways like IFN-γ and tertiary lymphoid structure (TLS) (Fig. 4A). Meanwhile, an elevated activation of TGF-β pathway was also observed, corroborating prior evidence for a beneficial role of TGF-β in ccRCC (Fig. 4A) [38]. In our previous findings, we have identified and validated dichotomous ccRCC immune subtypes with prognosis and TKI responses [39]. On this basis, we further explored whether the distribution of the TME-A/B subtypes differed across the PBRM1-RAD51 subgroups. A larger fraction of the TME-A subtype was observed in the dual-loss subgroup (*P* < 0.001; Fig. 4B). Except for PD-L1 status (*P* = 0.116; Fig. 4B), canonical checkpoint molecules were generally lower in dual-loss ccRCC (Supplementary Fig. 7).

Moreover, the immune cell populations of dual-loss ccRCC were characterized. A pronounced M1 polarization and low CD8^+^ T cells characterized dual-loss ccRCC. IHC results from ZSHS cohort validated M1 polarization (*P* = 0.041; Fig. 4D) and low CD8^+^ T cells infiltration of dual-loss ccRCC (*P* = 0.003; Fig. 4D). Though the significance in CD4^+^ T cells infiltration was absent in ZSHS cohort (*P* = 0.074), a higher Th1/Th2 proportion was observed (*P* = 0.048; Supplementary Fig. 8). Relatively low T cell receptor (TCR) Shannon diversity was observed in dual-loss and RAD51 single-loss ccRCC (*P* = 0.012; Fig. 4E). Meanwhile, a trend of elevated TCR evenness was observed in dual-loss ccRCC (*P* = 0.062; Fig. 4E). Dual-loss ccRCC also demonstrated the lowest MHC-II pathway expression (*P* = 0.037; Fig. 4F). Collectively, the tumor immune microenvironment (TIME) in dual-loss subgroup may be remodeled to a low-infiltrated TME but evoking effective immune-mediated tumor killing.

### Dual-loss of PBRM1 and RAD51 identifies distinct oncogenic and biological patterns

Oncogenic signaling pathways differ among individuals and significantly affect tumor progression and biological properties [2, 29]. Ten canonical pathways, including cell cycle, Hippo, Myc, Notch, Nrf2, PI3K-Akt, RTK-RAS, TGFβ, p53 and β-catenin/Wnt, were evaluated among subtypes [29]. PBRM1 and RAD51 dual-loss subtype exhibited higher alternation frequency of RTK-RAS pathways (Supplementary Fig. 9A). A recent analysis of the IMmotion151 trial identified several clusters with distinct biological pathways and responses to ICB (atezolizumab plus bevacizumab) therapy [40]. Of note, in dual-loss subtype, angiogenesis and fatty acid oxidation (FAO)/AMP-activated protein kinase (AMPK) pathways were activated while cell cycle and fatty acid synthesis (FAS)/pentose phosphate pathways were reduced, suggesting dual-loss ccRCC might harbor a reprogrammed fatty acid metabolism (Supplementary Fig. 9B).

### Dual-loss of PBRM1 and RAD51 ccRCC is susceptible to homologous recombination deficiency but possesses high chromosomal stability

**Fig. 5:**
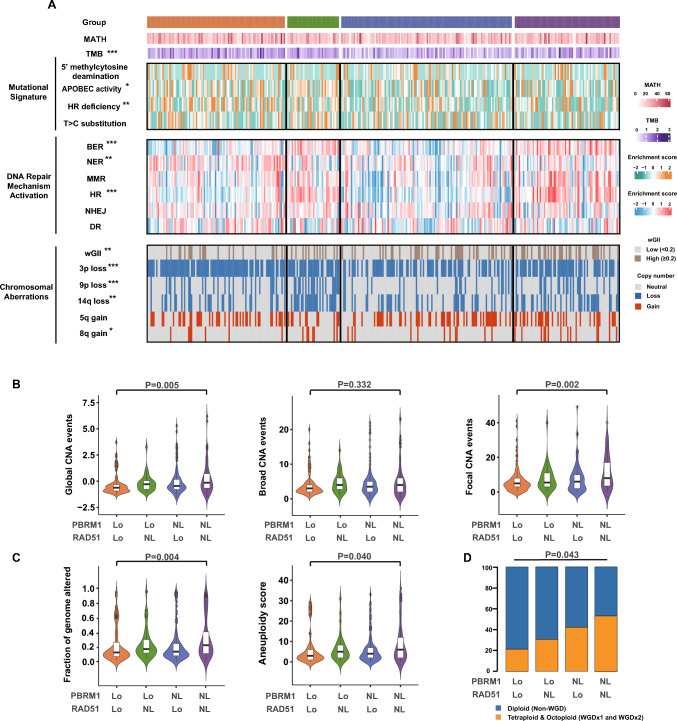
Dual-loss of PBRM1 and RAD51 ccRCC is susceptible to homologous recombination deficiency but possess high chromosomal stability. **A** Heatmap quantifying the activation level of different DDR mechanisms, mutational signatures and chromosomal aberrations in TCGA cohort. Data were analyzed by Kruskal–Wallis test and Pearson’s Chi-squared test. **B** Violin plot estimating the burden of global, broad and focal chromosomal alternations. Data were analyzed by Kruskal–Wallis test. **C** Estimation of chromosomal instability by calculating fraction of genome altered score (FGA) and aneuploidy score. Data were analyzed by Kruskal–Wallis test. **D** Bar charts showing the fraction of diploid, tetraploid and octoploid in each subgroup. Data were analyzed by Pearson’s Chi-squared test. *P ≤ 0.05; **P ≤ 0.01; ***P ≤ 0.001. *P* ≤ 0.05 was considered statistical significance. *Lo* Loss; *NL* Non-loss; *MATH* mutant-allele tumor heterogeneity; *BER* base excision repair; *NER* Nucleotide excision repair; *MMR* Mismatch repair; *HR* Homologous recombination; *NHEJ* Non-homologous end joining; *DR* Direct repair; *wGII* Weighted genome integrity index

To determine genomic features of dual-loss ccRCC, mutational signatures were studied using SigMA (Signature Multivariate Analysis algorithm) [23]. Of the identified signatures, signature HRD and APOBEC activity demonstrated increased levels in dual-loss ccRCC (*P* = 0.010 and *P* = 0.015 respectively; Fig. 5A). Further, base excision repair (BER) and homologous recombination repair (HR) pathway activities were downregulated (*P* < 0.001 and *P* < 0.001 respectively; Fig. 5A), while nucleotide excision repair (NER) was significantly upregulated in dual-loss subgroup (*P* = 0.004; Fig. 5A). These observations in mutational signatures and pathway activities were bolstered by similar significance in CheckMate cohort (Supplementary Fig. 10). Our results perhaps indicate that in dual-loss ccRCC, upregulated NER might offset BER deficiency while HR deficiency cannot be compensated by backup repairing pathways, mainly non-homologous end joining (NHEJ), leading to accumulating DSB errors [15].

DNA damage repair deficiencies were strongly associated with copy number variations in a large spectrum of cancers including hepatocellular carcinoma, non-small cell lung cancer and melanoma [9, 30, 41]. Here, we were intrigued that dual-loss ccRCC was highly specific for the lowest chromosomal aberrations in terms of fraction of genome altered (FGA), aneuploidy score and global CNA scores (*P* = 0.004, *P* = 0.040 and *P* = 0.005 respectively; Fig. 5B, C). It has been revealed that broad and focal CNAs, respectively, positively correlate with cancer cell proliferation and immune evasion [30]. Noteworthy, dual-loss ccRCC featured the lowest focal CNAs (*P* = 0.002), but not broad CNAs (*P* = 0.332), suggesting low occurrence of immune evasion but not tumor proliferation in dual-loss subgroup (Fig. 5B). Weighted genome integrity index (wGII), which has previously been associated with a more aggressive phenotype in ccRCC [42], was also the lowest in dual-loss ccRCC (*P* = 0.002). We detected that though harboring highest frequency of 3p loss, dual-loss subtype harbors lowest frequency of 9p loss, 14q loss and 8q gain, which were among the most common copy number alterations (*P* < 0.001, *P* = 0.002 and *P* = 0.037 respectively; Fig. 5A) [4]. Moreover, the proportion of tetraploid and octoploid was the lowest in dual-loss ccRCC (*P* = 0.043; Fig. 5D). Thus, we came to the conclusion that dual-loss subgroup features defective HR status but the highest chromosomal stability.

## Discussion

In the setting of ccRCC, no biomarker predicting response of immunotherapy has been used in clinical practice currently [2, 8, 40]. Frequently investigated biomarkers in other cancer types were found insufficient to meet the need for accurate prediction. DDR-related biomarkers, whose value of predicting immunotherapeutic responses has been confirmed in various solid tumors [8, 13], seem to be promising novel biomarkers meeting the therapeutic need. However, precursory methods using single point-mutation-based detecting methods could only identify a paucity of patients vulnerable to ICB (less than 20%) since few ccRCCs were characterized by alternations in DDR pathways [2]. Herein, we illustrated a HRD function-based PBRM1-RAD51 classification capable of predicting responses to immunotherapy.

While the role of PBRM1 status as a predicator of immunotherapy remains to be validated, PBRM1-mutant ccRCC seems to present a distinct nonimmunogenic phenotype from PBRM1-wild type ccRCC [12]. On the other hand, the coordination between PBRM1 loss and increased replication stress, DSB and chromosomal instability has been validated by previous publications [9, 10]. PARP1, mainly involved in BER, has been linked with immunotherapy responses in PBRM1-mutant ccRCC [43]. Bolstered by previous efforts across multiple contexts, we demonstrated that the impaired DNA double-strand break of PBRM1 status mainly lies in the affected HR with RAD51 loss of expression as a feature, while NHEJ remains largely unaffected.

Mounting evidence has established HRD-associated RAD51 as a key therapeutic biomarker to chemotherapy and other DDR inhibitors [16, 17, 19]. In ESCC (esophageal squamous cell carcinoma), RAD51 expression indicated responses to chemotherapy [17]. Moreover, PARP inhibitor (PARPi) benefit was observed in prostate cancer [20] and breast cancer [16] with loss of RAD51 foci. Recent efforts have used tumor immune dysfunction and exclusion (TIDE), a mRNA expression-based algorithm, linking RAD51 expression to potential immunotherapeutic responses [44]. However so far, a paucity of studies linked patient-level immunotherapeutic outcomes with RAD51 status. Our study sheds light on the predictive value of HRD-associated RAD51 in prognosis and responsiveness to immunotherapy in ccRCC. Additionally, a highly HRD ccRCC subtype, featured as PBRM1 and RAD51 loss, was found hyper-sensitive to immunotherapy.

In our study, we highlighted that dual-loss of PBRM1 and RAD51 delineates a specific ICB-sensitive ccRCC subset with enriched M1 macrophages. Of particular interest is that dual-loss ccRCC does not exhibit previously presumed indicators of immunotherapy sensitivity including TLS, which might be attributed to the heterogeneous TLS localization. It has been demonstrated that heterogeneity of TLS correlated with macrophage polarization and different clinical outcomes [45, 46]. Our study further imparts insights that incorporating genomic events into the analysis of deconstructing TME constitutes entails consideration as many levels of studies have also linked genomic events to the remodeling of immune contexture [47].

Our study also found that PBRM1 and RAD51 dual-loss patients are highly homologous recombination deficient, which implies potential sensitivity for PARPi. Furthermore, several on-going clinical trials have focused on PARPi in ccRCC (NCT03741426, NCT03207347). Potential application of PARPi in dual-loss ccRCC subtype raised by our research may require further investigation.

Limitations of this study should be taken into consideration. Firstly, its retrospective nature brings forth the possibility of introducing biases related to sampling and batch effects. Furthermore, the inclusion of diverse cohorts with varying baseline characteristics may potentially engender inherent biases. Additionally, owing to the relatively limited number of patients in ZSHS-ICB, we had to recognize the absence of statistical differences between subgroups regarding information like irAEs, and more validation is needed to enhance the credibility of our study.

In conclusion, our study identifies a specific highly homologous recombination defective ccRCC subtype, featured as PBRM1 and RAD51 dual-loss. Dual-loss ccRCC is hyper-sensitive to immunotherapy. Though dual-loss subtype is susceptible to defective homologous recombination, it possesses high chromosomal stability. Besides, this subtype harbors an immune-desert but effective microenvironment with enriched M1 cells.

### Supplementary Information

Below is the link to the electronic supplementary material.

Supplementary file1 (DOCX 2612 KB)

## Data Availability

Data and materials generated that are relevant to the results are included in this article. Other data are available from the corresponding author Prof. Xu upon reasonable request.

## Abbreviations

AMPK: AMP-activated protein kinase
BER: Base excision repair
ccRCC: Clear cell renal cell carcinoma
CNA: Copy number alternation
DDR: DNA damage repair
DSB: Doubles strand break
FAO: Fatty acid oxidation
FAS: Fatty acid synthesis
FGA: Fraction of genome altered
HR: Homologous recombination/hazard ratio
HRD: Homologous recombination deficiency
ICB: Immune checkpoint blockade
IHC: Immunohistochemistry
Lo: Loss
mRCC: Metastatic renal cell carcinoma
NER: Nucleotide excision repair
NHEJ: Non-homologous end joining
OS: Overall survival
PARPi: Poly (ADP-ribose) polymerase inhibitor
PD-L1: Programmed cell death ligand 1
PBRM1: Polybromo 1
RCC: Renal cell carcinoma
SigMA: Signature multivariate analysis algorithm
TCGA: The cancer genome atlas
TCR: T cell receptor
TMA: Tissue microarray
TMB: Tumor mutation burden
TIDE: Tumor immune dysfunction and exclusion algorithm
TIME: Tumor immune microenvironment
TKI: Tyrosine kinase inhibitors
WES: Whole-exome sequencing
ZSHS: Zhongshan hospital
