# Supplementary material for: Dual-loss of PBRM1 and RAD51 identifies hyper-sensitive subset patients to immunotherapy in clear cell renal cell carcinoma
Source: Cancer Immunol Immunother. 2024 Apr 12;73(5):95. doi: 10.1007/s00262-024-03681-x (PMC11014826; doi:10.1007/s00262-024-03681-x)
Supplement: Supplementary file 1 — Supplementary file1 (DOCX 2612 KB) [file 262_2024_3681_MOESM1_ESM.docx]

**Dual-loss of PBRM1 and RAD51 identifies hyper-sensitive subset patients to immunotherapy in clear cell renal cell carcinoma**

**Running Title: Dual-loss of PBRM1 and RAD51 in ccRCC**

Ziyang Xu^1,3^, Wenbin Jiang^1,3^, Li Liu^1,3^, Youqi Qiu^1^, Jiahao Wang^1^, Siyuan Dai^1^, Jianming Guo^1, *^, Jiejie Xu^2,*^

^1^Department of Urology, Zhongshan Hospital, Fudan University, Shanghai, China;

^2^NHC Key Laboratory of Glycoconjugate Research, Department of Biochemistry and Molecular Biology, School of Basic Medical Sciences, Fudan University, Shanghai, China;

^3^These authors contributed equally as first authors.

**Supplementary Materials**

**Supplementary Figure 1. Flow chart of cohort selection.**

**Supplementary Figure 2. Representative images of immunohistochemistry.**

**Supplementary Figure 3. Distribution of RAD51 expression.**

**Supplementary Figure 4. The association of disease progression, risk categorization and PBRM1-RAD51 status.**

**Supplementary Figure 5. Multivariate Cox regression in ZSHS-ICB cohort.** **Supplementary Figure 6. Univariable Cox regression analysis showing the predictive value of PBRM1-RAD51 versus various ICB candidate biomarkers.**

**Supplementary Figure 7. Canonical checkpoint molecules expression among PBRM1-RAD51 subgroups.**

**Supplementary Figure 8. The immune contexture among PBRM1-RAD51 subgroups in ZSHS cohort.**

**Supplementary Figure 9. The oncogenic and biological patterns among PBRM1-RAD51 subgroups.**

**Supplementary Figure 10. The association of DDR pathway activation and mutational signatures in CheckMate cohort.**

**Supplementary Table 1. Clinicopathological parameters in ZSHS cohort.**

**Supplementary Table 2. Clinicopathological parameters in ZSHS-ICB cohort.**

**Supplementary Table 3. Distribution of PBRM1-RAD51 status across cohorts.**

**Supplementary Table 4. Immune-related adverse events in ZSHS-ICB cohort.**

**Supplementary Table 5. Gene signature and data availability.**

**Supplementary Table 6. Immunohistochemistry (IHC) antibodies.**


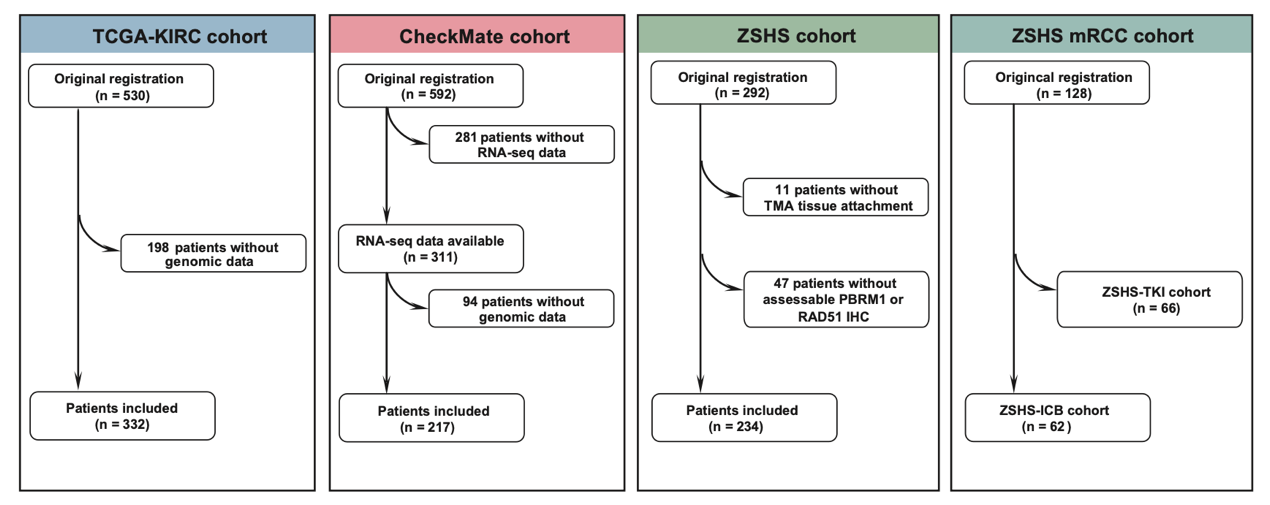


**Supplementary Figure 1. Flow chart of cohort selection.** Inclusion and exclusion criteria for two public datasets and local institutional cohorts (ZSHS cohort and ZSHS-mRCC cohort) included in this study.


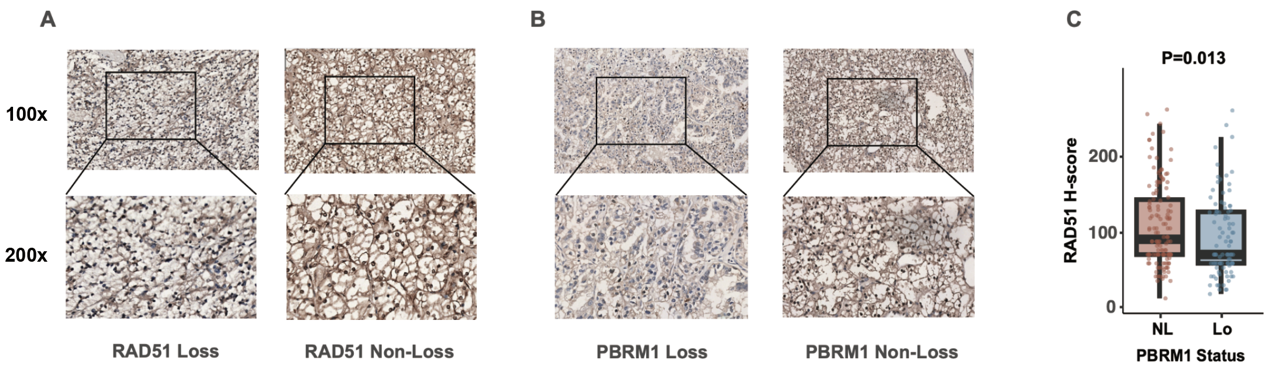


**Supplementary Figure 2. Representative images of immunohistochemistry.** **(A-B)** Representative images of Immunohistochemistry (IHC) of **(A)** RAD51 and **(B)** PBRM1 in the tissue of clear cell renal cell carcinoma. **(C)** The relationship of RAD51 status and PBRM1 IHC H-score in ZSHS and ZSHS-mRCC cohorts. P ≤ 0.05 was considered statistical significance. Lo, Loss; NL, Non-loss.


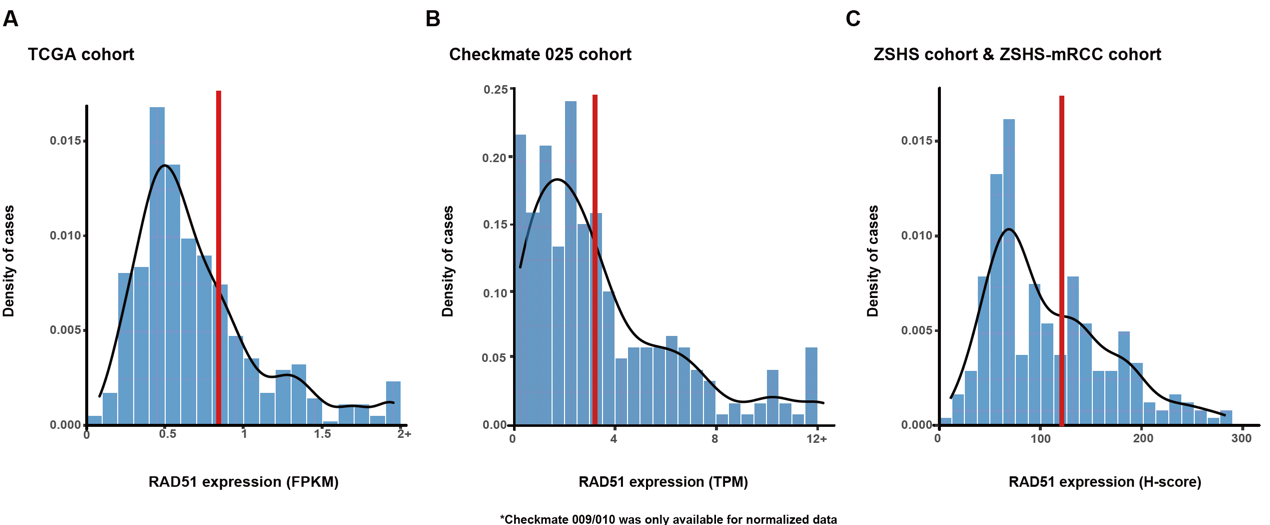


**Supplementary Figure 3. Distribution of RAD51 expression.** Distribution of RAD51 expression in **(A)** TCGA, **(B)** CheckMate and **(C)** ZSHS and ZSHS-mRCC cohorts. Lo, Loss; NL, Non-loss.


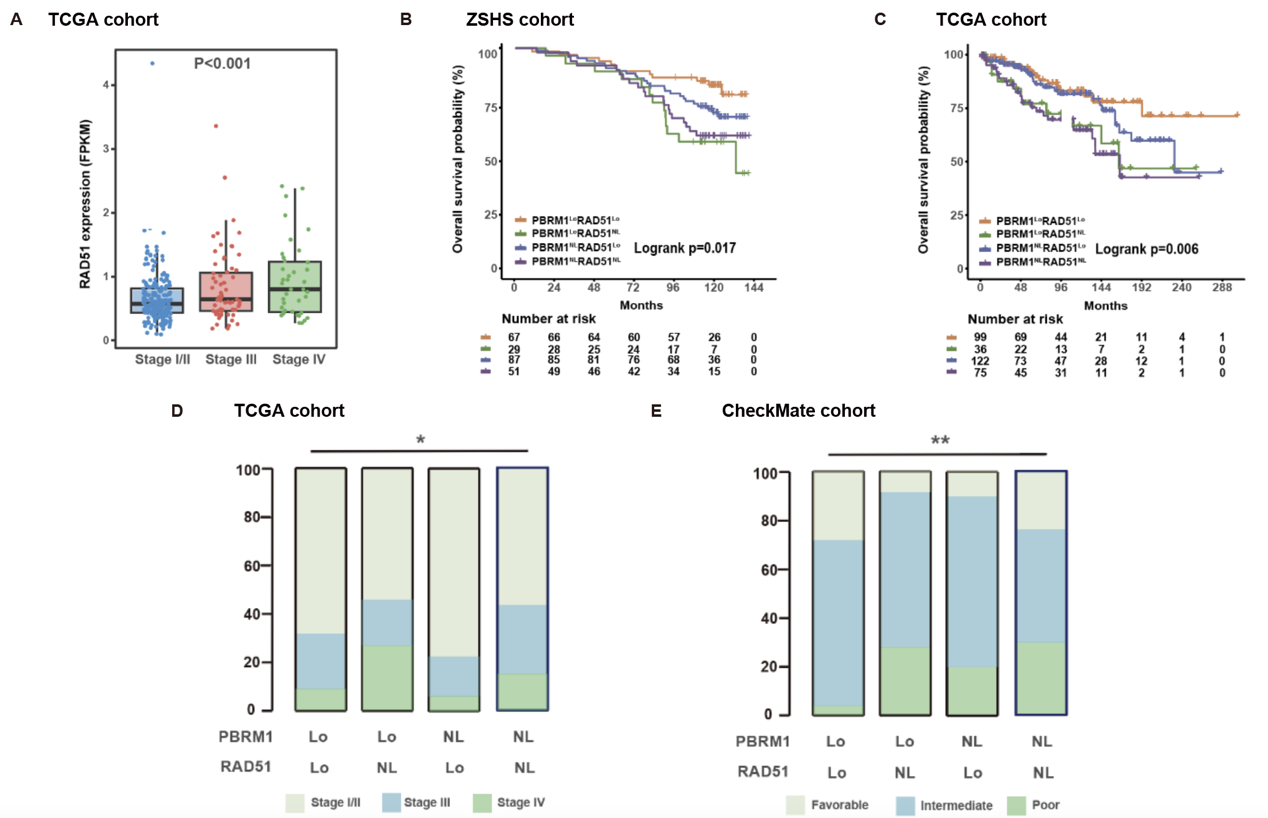


**Supplementary Figure 4. The association of disease progression, risk categorization and PBRM1-RAD51 status. (A)** The association of RAD51 expression and TNM stages in TCGA cohort. **(B)** The association of PBRM1-RAD51 status and prognosis in ZSHS cohort. **(C)** The association of PBRM1-RAD51 status and prognosis in TCGA cohort. **(D)** The association of PBRM1-RAD51 status and TNM stages in TCGA cohort. **(E)** The association of PBRM1-RAD51 status and International Metastatic Renal Cell Carcinoma Database Consortium (IMDC) risk stages in CheckMate cohort. Data were analyzed by Kruskal-Wallis Test and Pearson’s chi-squared test. *, P ≤ 0.05; **, P ≤ 0.01; ***, P ≤0.001. P ≤ 0.05 was considered statistical significance. Lo, Loss; NL, Non-loss.


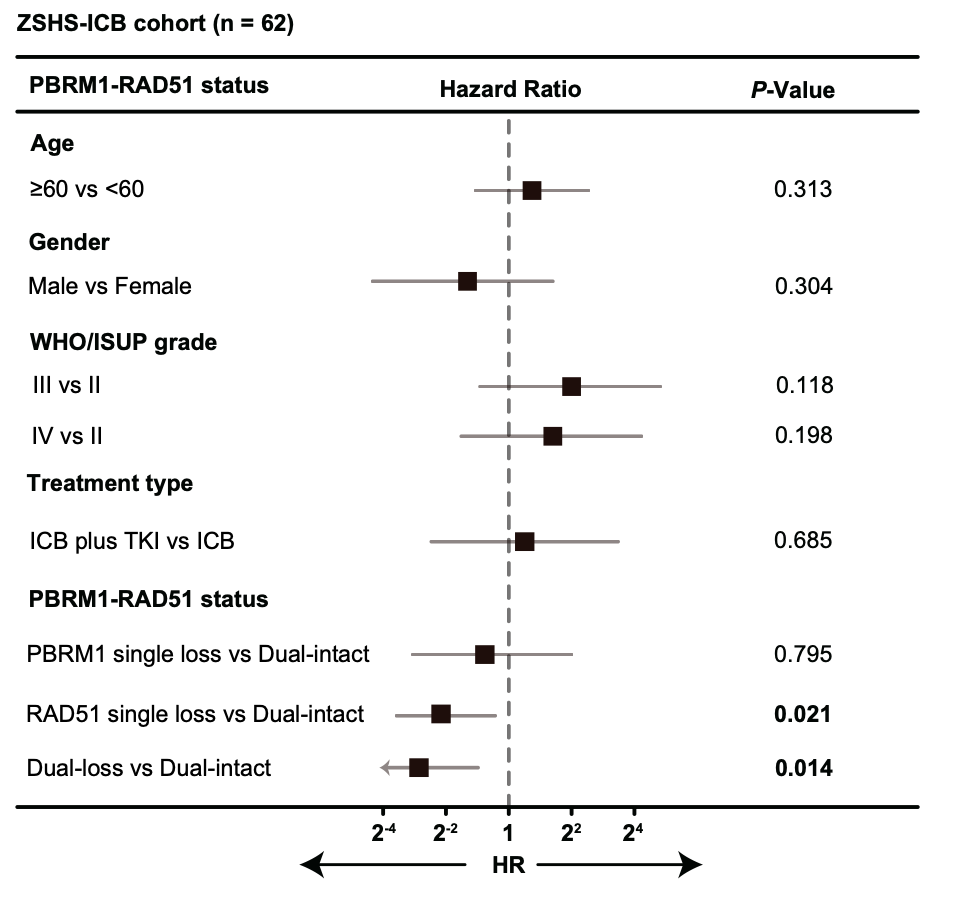


**Supplementary Figure 5. Multivariate Cox regression in ZSHS-ICB cohort.** Multivariate Cox regression in ZSHS-mRCC cohort with the inclusion of clinicopathological factors including age, gender, treatment types and tumor grades (WHO/ISUP)**.** P ≤ 0.05 was considered statistical significance.

**
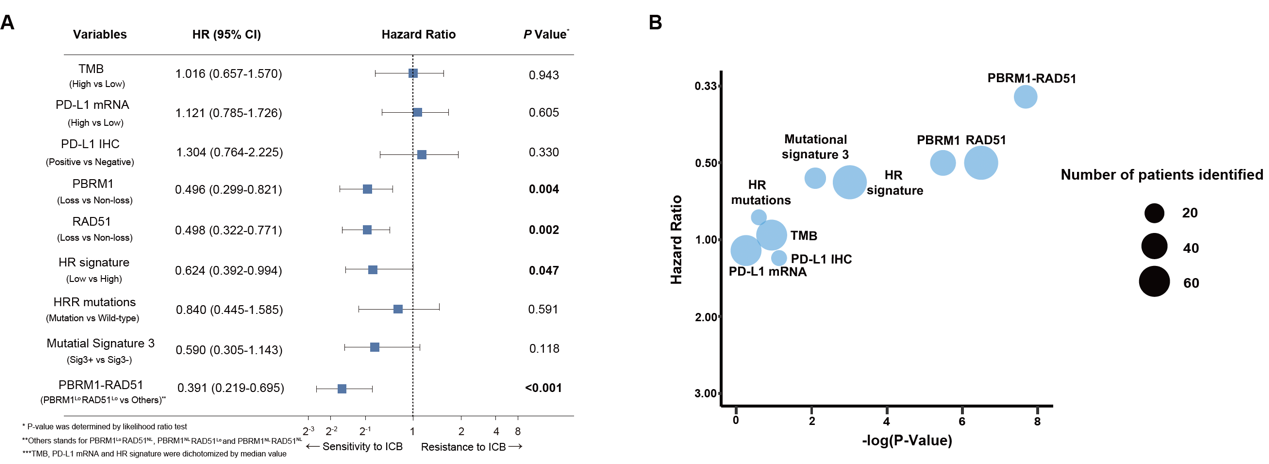
**

**Supplementary Figure 6.** **Univariable Cox regression analysis showing the predictive value of** **PBRM1-RAD51 versus various ICB candidate biomarkers. (A)** Table indicating the predictivity of various ICB candidate biomarkers to ICB responses by univariate cox regression model. **(B)** P-log (HR) plot demonstrating the predictivity of PBRM1-RAD51 status outperformed various ICB candidate biomarkers to ICB responses. P ≤ 0.05 was considered statistical significance. Lo, Loss; NL, Non-loss.

**
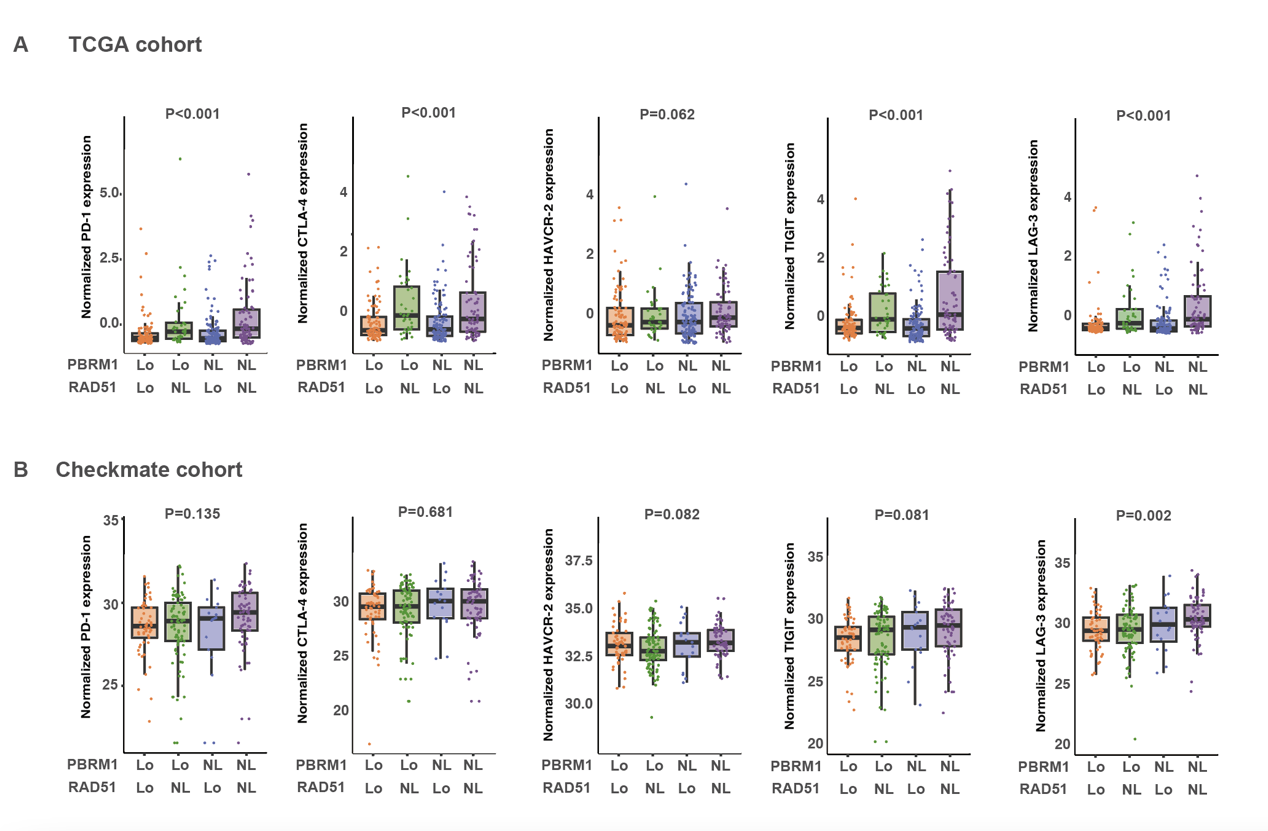
**

**Supplementary Figure 7. Canonical checkpoint molecules expression among PBRM1-RAD51 subgroups.** Evaluation of PD-1, CTLA-4, HAVCR-2, TIGIT, LAG-3 among PBRM1-RAD51 subtypes in **(A)** TCGA cohort and **(B)** CheckMate cohort. Data were analyzed by Kruskal-Wallis test. P ≤ 0.05 was considered statistical significance. Lo, Loss; NL, Non-loss.

**
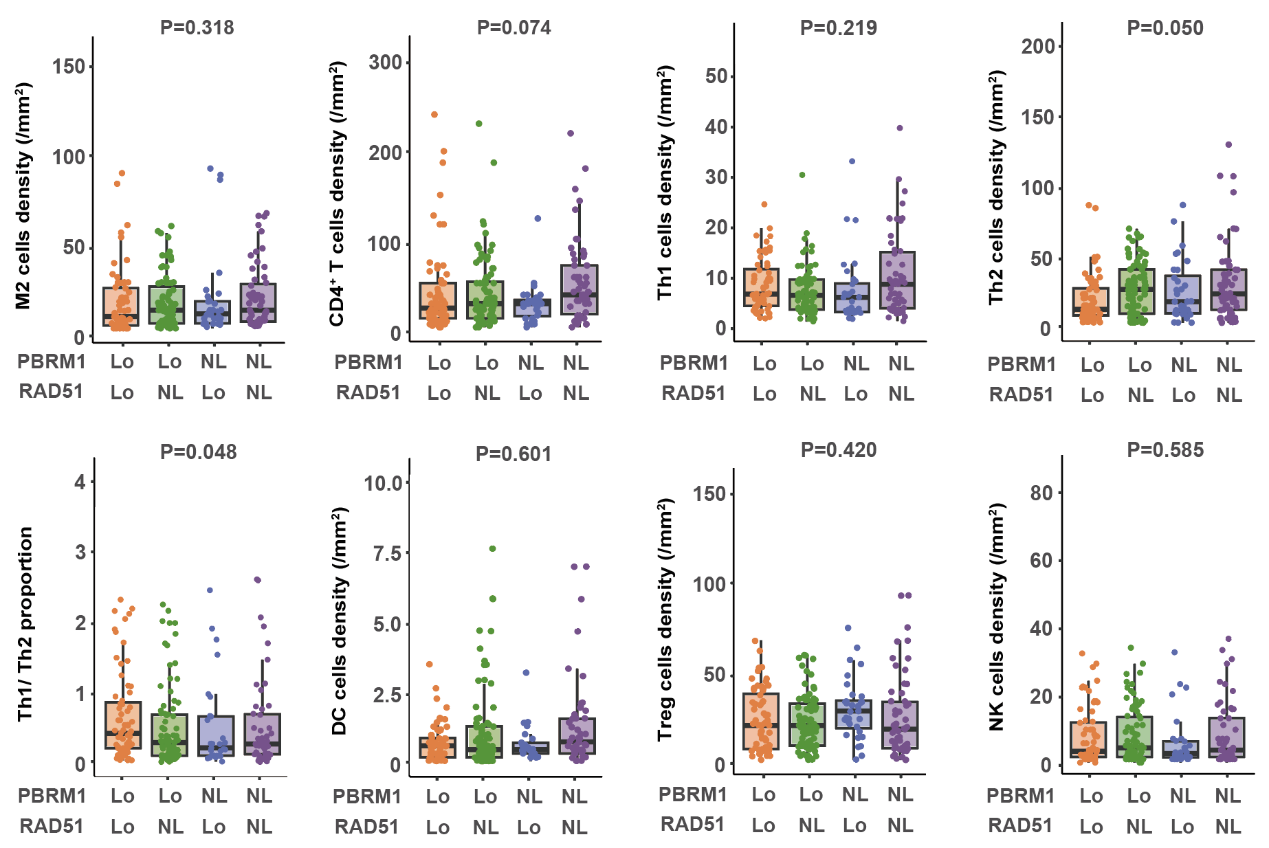
**

**Supplementary Figure 8. The immune contexture** **among PBRM1-RAD51 subgroups in ZSHS cohort.** Evaluation of M2 cells, CD4^+^T cells, Th1 cells, Th2 cells, DC cells, Th1 /Th2 proportion, Treg cells and NK cells among PBRM1-RAD51 subtypes by IHC. Data were analyzed by Kruskal-Wallis test. P ≤ 0.05 was considered statistical significance. Lo, Loss; NL, Non-loss.

**
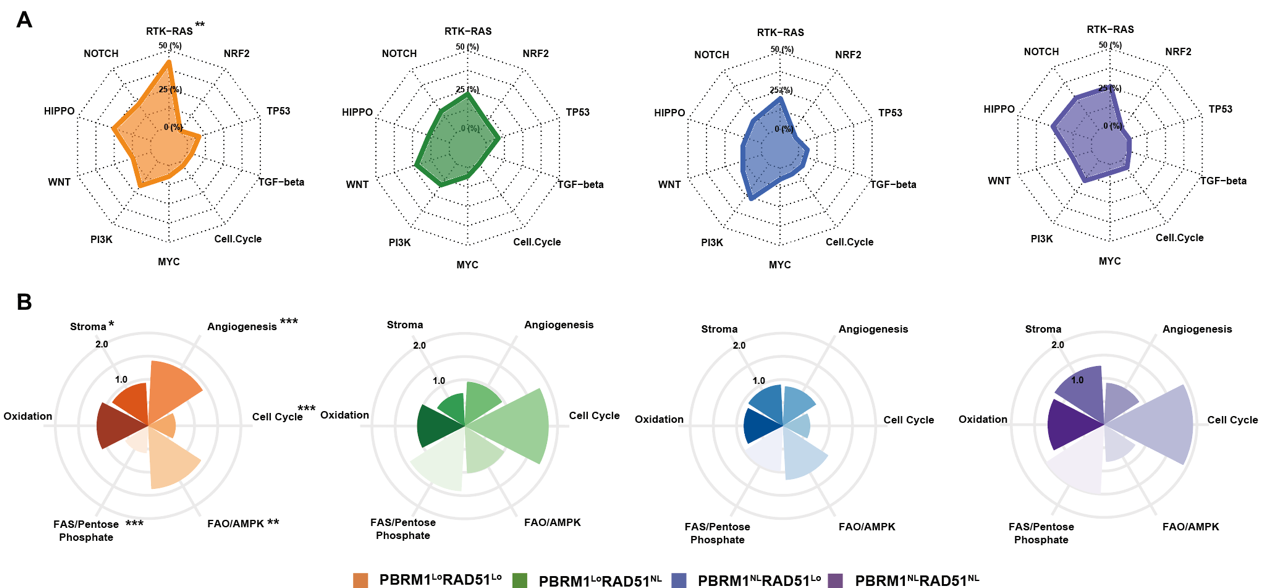
**

**Supplementary Figure 9. The oncogenic and biological patterns among PBRM1-RAD51 subgroups. (A)** Radar chart demonstrating the percentage of patients with alternations in the corresponding oncogenic pathways among different subgroups in TCGA cohort. **(B)** Circular bar plot demonstrating the level of stroma, angiogenesis, cell cycle, FAO/AMPK, FAS/Pentose Phosphate and complement/Ω-oxidation among different subgroups in TCGA cohort. Data were analyzed by Kruskal-Wallis test. *, P ≤ 0.05; **, P ≤ 0.01; ***, P ≤0.001. P ≤ 0.05 was considered statistical significance. Lo, Loss; NL, Non-loss; FAO, fatty acid oxidation; AMPK, AMP-activated protein kinase; FAS, fatty acid synthesis.

**
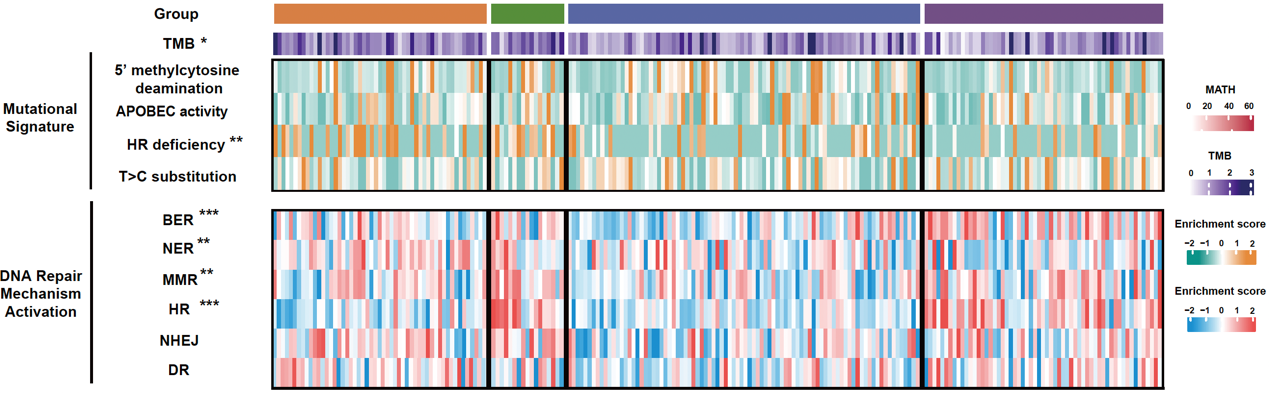
**

**Supplementary Figure 10. The association of DDR pathway activation and mutational signatures in CheckMate cohort.** Heatmap quantifying the activation level of different DDR mechanisms and mutational signatures in CheckMate cohort. Data were analyzed by Kruskal-Wallis test. *, P ≤ 0.05; **, P ≤ 0.01; ***, P ≤0.001. P ≤ 0.05 was considered statistical significance. Lo, Loss; NL, Non-loss. MATH, mutant-allele tumor heterogeneity. BER, base excision repair. NER, nucleotide excision repair. MMR, mismatch repair. HR, homologous recombination. NHEJ, non-homologous end joining. DR, direct repair.

| **Supplementary Table 1. Clinicopathological parameters in ZSHS cohort.** | | | | | | |
| --- | --- | --- | --- | --- | --- | --- |
| **Parameters** | **ZSHS Cohort (*n* = 234)** | | | | | |
|  | **No** | PBRM1^Lo^  RAD51^Lo^  (*n* = 67) | PBRM1^Lo^  RAD51^NL^  (*n* = 29) | PBRM1^NL^  RAD51^Lo^  (*n* = 87) | PBRM1^NL^  RAD51^NL^  (*n* = 51) | ***P*** |
| **Total** | 234 | 67 | 29 | 87 | 51 |  |
| **Age** |  |  |  |  |  | 0.097 |
| ≥50 | 150 | 35 | 20 | 59 | 36 |  |
| **<**50 | 84 | 32 | 9 | 28 | 15 |  |
| **Gender** |  |  |  |  |  | 0.938 |
| Male | 164 | 45 | 21 | 62 | 36 |  |
| Female | 70 | 22 | 8 | 25 | 15 |  |
| **ECOG PS** |  |  |  |  |  | 0.190 |
| 0 | 171 | 49 | 16 | 67 | 39 |  |
| 1 | 53 | 15 | 10 | 19 | 9 |  |
| ≥2 | 10 | 3 | 3 | 1 | 3 |  |
| **pT stage** |  |  |  |  |  | 0.721 |
| I | 160 | 45 | 20 | 61 | 34 |  |
| II | 25 | 5 | 2 | 10 | 8 |  |
| III/IV | 49 | 17 | 7 | 16 | 9 |  |
| **Necrosis** |  |  |  |  |  | 0.471 |
| Absent | 205 | 58 | 27 | 78 | 42 |  |
| Present | 29 | 9 | 2 | 9 | 9 |  |
| **Events** |  |  |  |  |  | **0.021** |
| Death | 71 | 12 | 26 | 13 | 20 |  |
| Alive | 163 | 55 | 61 | 16 | 31 |  |

Abbreviations: AJCC = American Joint Committee on Cancer; ECOG PS = Eastern cooperative oncology Group performance status; pT stage=Pathological stage

*P value was used from Pearson’s chi-square test, significant P value ≤ 0.05 was shown in bold.

| Supplementary Table 2. Clinicopathological parameters in ZSHS-ICB cohort. | | | | | | |
| --- | --- | --- | --- | --- | --- | --- |
| Parameters | **ZSHS-ICB Cohort (*n* = 62)** | | | | | |
|  | **No** | PBRM1^Lo^  RAD51^Lo^  (*n* = 20) | PBRM1^Lo^  RAD51^NL^  (*n* = 10) | PBRM1^NL^  RAD51^Lo^  (*n* = 21) | PBRM1^NL^  RAD51^NL^  (*n* = 11) | ***P*** |
| Age |  |  |  |  |  | 0.473 |
| ≥60 | 36 | 9 | 6 | 13 | 8 |  |
| <60 | 26 | 11 | 4 | 8 | 3 |  |
| Gender |  |  |  |  |  | 0.884 |
| Male | 46 | 14 | 7 | 16 | 9 |  |
| Female | 16 | 6 | 3 | 5 | 2 |  |
| Stages |  |  |  |  |  | **0.041** |
| II | 29 | 16 | 3 | 7 | 3 |  |
| III | 18 | 1 | 4 | 8 | 5 |  |
| IV | 8 | 2 | 2 | 3 | 1 |  |
| NA | 7 | 1 | 1 | 3 | 2 |  |
| Treatment types |  |  |  |  |  | 0.263 |
| ICB | 12 | 2 | 1 | 5 | 4 |  |
| ICB plus TKI | 50 | 18 | 9 | 16 | 7 |  |
| Events |  |  |  |  |  | 0.052 |
| Death | 10 | 1 | 4 | 2 | 3 |  |
| Alive | 52 | 19 | 6 | 19 | 8 |  |

Abbreviations: ICB = Immune checkpoint blockade; TKI = Tyrosine kinase inhibitors.

*P value was used from Pearson’s chi-square test, significant P value ≤ 0.05 was shown in bold.

| **Supplementary Table 3.** **Distribution of PBRM1-RAD51 status across cohorts.** | | | | | |
| --- | --- | --- | --- | --- | --- |
| **Parameters** | **Combined Cohort (*n* = 911)** | | | | |
|  | **Patients**  **No** | PBRM1^Lo^  RAD51^Lo^ | PBRM1^Lo^  RAD51^NL^ | PBRM1^NL^  RAD51^Lo^ | PBRM1^NL^  RAD51^NL^ |
| Total | 911 | 264 (29.0%) | 101 (11.1%) | 361 (39.6%) | 205 (22.5%) |
| ZSHS | 234 | 67 (28.6%) | 29 (12.4%) | 87 (37.1%) | 51 (21.8%) |
| ZSHS-mRCC | 128 | 45 (35.2%) | 19 (14.8%) | 42 (32.8%) | 22 (17.2%) |
| TCGA | 332 | 99 (29.8%) | 36 (10.8%) | 122 (36.7%) | 75 (22.6%) |
| CheckMate | 217 | 53 (24.4%) | 17 (7.8%) | 110 (50.7%) | 57 (26.3%) |

| Supplementary Table 4. Immune-related adverse events (irAEs) in ZSHS-ICB cohort. | | | | | |
| --- | --- | --- | --- | --- | --- |
| Parameters | **ZSHS-ICB Cohort (*n* = 52)** | | | | |
|  | **No** | PBRM1^Lo^  RAD51^Lo^  (*n* = 17) | PBRM1^Lo^  RAD51^NL^  (*n* = 10) | PBRM1^NL^  RAD51^Lo^  (*n* = 15) | PBRM1^NL^  RAD51^NL^  (*n* = 10) |
| Any event | 39 | 14 | 8 | 11 | 6 |
| Hyper-tension | 14 | 5 | 2 | 4 | 3 |
| Asthenia | 12 | 5 | 2 | 2 | 3 |
| Proteinuria | 5 | 0 | 1 | 2 | 1 |
| Back pain | 5 | 3 | 0 | 1 | 1 |
| Increased ALT or  AST levels | 5 | 1 | 0 | 3 | 1 |
| Hypothyroidism | 4 | 1 | 1 | 2 | 0 |
| Diarrhea | 4 | 2 | 1 | 0 | 1 |
| Mucosal inflammation | 4 | 0 | 0 | 1 | 3 |
| Increased blood creatine | 4 | 2 | 1 | 0 | 1 |
| Decreased appetite | 2 | 1 | 0 | 0 | 1 |
| Rash | 2 | 0 | 2 | 0 | 0 |

| **Supplementary Table 5. Gene signature and data availability.** |
| --- |

| Signature and data | | Gene | Source |
| --- | --- | --- | --- |
| Suppression score | *CD274, IDO1, FASLG, CTLA4, PDCD1, LAG3, HAVCR2, PDCD1LG2, IL10, TGFB1, PTGS2* | | PMID:27699256 |
| MHC- I | *HLA-A, HLA-B, HLA-C, B2M, TAP1, TAP2, TAPBP* | | PMID: 34019806 |
| MHC- II | *HLA-DRA, HLA-DRB1, HLA-DMA, HLA-DPA1, HLA-DPB1, HLA-DMB, HLA-DQB1, HLA-DQA1, CIITA* | | PMID: 34019806 |
| IFNG-related gene signature* | *CD8A, CCL5, CD27, CD274, PDCD1LG2, CD276, CMKLR1, CXCL9, CXCR6, HLA-DQA1, HLA-DRB1, HLA-E, IDO1, LAG3, NKG7, PSMB10, STAT1, TIGIT* | | PMID: 28650338 |
| Tertiary Lymphoid Structure | *CCL2, CCL3, CCL4, CCL5, CCL8, CCL18, CCL19, CCL21, CXCL9, CXCL10, CXCL11, CXCL13* | | PMID: 23097687 |
| TGF-[β](http://www.baidu.com/link?url=AKrYylZaMCNwJe7PL-8yaANd0wqLdarHDTPb4ER5XGWPDVS4F2XBqfxfOFqdvuWNBUDofPAV8TOQfGUgfWxWLqA2ILVkL30a88ShLyoiFC3) related signature | *ACVR1, APC, ARID4B, BCAR3, BMP2, BMPR1A,* *CDH1, CDK9, CDKN1C, CDKN1C, CTNNB1, ENG, FKBP1A, FNTA, FURIN, HDAC1, HIPK2, ID1, ID2, ID3, IFNGR2, IFNGR2, JUNB, KLF10, LEFTY2, LTBP2, MAP3K7, NCOR2, NOG, PMEPA1, PPM1A, PPP1CA, PPP1R15A, RAB31, RHOA, SERPINE1, SKI, SKIL, SLC20A1, SMAD1, SMAD3, SMAD6, SMAD7, SMURF1, SMURF1, SMURF2, SPTBN1, TGFB1, TGFBR1, TGIF1, THBS1, TJP1, TJP1, TRIM33, UBE2D3, WWTR1, XIAP* | | Msigdbr database |
| Base excision repair | *PARP1, POLB, APEX1, APEX2, FEN1, TDG, TDP1, UNG* | | PMID:29617664 |
| Homologous recombination | *MRE11A, NBN, RAD50, TP53BP1, XRCC2, XRCC3, BARD1, BLM, BRCA1, BRCA2, BRIP1, EME1, GEN1, MUS81, PALB2, RAD51, RAD52, RBBP8, SHFM1, SLX1A, TOP3A* | | PMID:29617664 |
| Nucleotide Excision Repair | *CUL5, ERCC1, ERCC2, ERCC4, ERCC5, ERCC6, POLE, POLE3, XPA, XPC* | | PMID:29617664 |
| Non-homologous End Joint | *LIG4, NHEJ1, POLL, POLM, PRKDC, XRCC4, XRCC5, XRCC6* | | PMID:29617664 |
| Mismatch Repair | *EXO1, MLH1, MLH3, MSH2, MSH3, MSH6, PMS1, PMS2* | | PMID:29617664 |
| Direct Repair | *ALKBH2, ALKBH3, MGMT* | | PMID:29617664 |
| T-effector | *CD8A, IFNG, EOMES, PRF1, CD274* | | PMID:33157048 |
| FAO/AMPK | *CPT2, PPARA, CPT1A, PRKAA2, PDK2, PRKAB1* | | PMID:33157048 |
| FAS/Pentose Phosphate | *FASN, PARP1, ACACA, G6PD, TKT, TALDO1, PGD* | | PMID:33157048 |
| Angiogenesis | VEGFA, KDR, ESM1, PECAM1, ANGPTL4, CD34 | | PMID:33157048 |
| Cell Cycle | *CDK2, CDK4, CDK6, BUB1B, CCNE1, POLQ, AURKA, MKI67, CCNB2* | | PMID:33157048 |
| Stroma | *FAP, FN1, COL5A1, COL5A2, POSTN, COL1A1, COL1A2, MMP2* | | PMID:33157048 |
| Immune score  calculated by ESTIMATE | */* | | PMID: 24113773 |
| Mutational signature 3  calculated by SigMA | */* | | PMID:30988514 |
| Aneuploidy score | */* | | PMID: 29617664 |

**Supplementary Table 6. Immunohistochemistry (IHC) antibodies.**

| No. | Antibody name | Clonality Species | Company | Product No. | Diluted | IHC |
| --- | --- | --- | --- | --- | --- | --- |
| 1 | Anti-PBRM1 antibody | Monoclonal Rabbit Anti-human | Bethyl Laboratories | A301-591A | 1:2000 | PBRM1 |
| 2 | Anti-RAD51 antibody | Monoclonal Rabbit Anti-human | Abcam | ab133534 | 1:300 | RAD51 |
| 3 | Anti-CD8 alpha antibody | Monoclonal Mouse Anti-human | Abcam | ab199016 | 1:400 | CD8^+^ T cells |
| 4 | Anti-CD4 antibody | Monoclonal Rabbit Anti-human | Abcam | ab213215 | 1:50 | CD4^+^ T cells |
| 5 | Anti-T-bet antibody | Monoclonal Mouse Anti-human | Abcam | ab91109 | 1:500 | Th1 (Type 1 helper T cells) |
| 6 | Anti-CD4 antibody | Monoclonal Rabbit Anti-human | Abcam | ab213215 | 1:50 |  |
| 7 | Anti-GATA3 antibody | Monoclonal Mouse Anti-human | Abcam | ab77073 | 1:200 | Th2 (Type 2 helper T cells) |
| 8 | Anti-CD4 antibody | Monoclonal Rabbit Anti-human | Abcam | ab213215 | 1:50 |  |
| 9 | Anti-FOXP3 antibody | Monoclonal Mouse Anti-human | Abcam | ab22510 | 1:1000 | Tregs (Regulatory T cells) |
| 10 | Anti-NCAM1 antibody | Monoclonal Mouse Anti-human | Abcam | ab233944 | 1:1000 | NKs (Natural killer cells) |
| 11 | Anti-CD11c antibody | Monoclonal Rabbit Anti-human | Abcam | ab52632 | 1:500 | DCs (Dendritic cells) |
| 12 | Anti-CD86 antibody | Monoclonal Rabbit Anti-human | Abcam | ab53004 | 1:500 | M1-polarized macrophages |
| 13 | Anti-CD163 antibody | Monoclonal Mouse Anti-human | Abcam | ab111250 | 1:50 | M2-polarized macrophages |
